# Unveiling Brevibacterium Species Isolated in the Cerebrospinal Fluid: A Report of a Rare Case

**DOI:** 10.7759/cureus.61072

**Published:** 2024-05-25

**Authors:** Julie Nguyen, Priyasheelta Nand

**Affiliations:** 1 Internal Medicine, St. Joseph's Medical Center, Stockton, USA; 2 Internal Medicine/Infectious Disease, St. Joseph's Medical Center, Stockton, USA

**Keywords:** infectious disease medicine, cerebrospinal fluid (csf), pulmonary adenocarcinoma, hiv aids, meningitis, brevibacterium

## Abstract

This case emphasizes the significance of recognizing and managing *Brevibacterium* species. Here, we present a unique case of *Brevibacterium* species isolated from the cerebrospinal fluid of a 60-year-old female with recently diagnosed human immunodeficiency virus (HIV) and small cell carcinoma of the lung. Management involved a two-week course of intravenous vancomycin. *Brevibacterium* species are infrequently encountered in clinical practice. Sharing this case report aims to enhance the limited understanding of *Brevibacterium* species infections and encourages discussion among healthcare professionals regarding its diagnosis and management.

## Introduction

*Brevibacterium* species are gram-positive, non-spore-forming bacteria commonly found in soil, dairy products, and human skin flora. They are considered opportunistic pathogens, typically associated with bloodstream infections in immunocompromised individuals. The presence of *Brevibacterium* species in the cerebrospinal fluid (CSF) is exceedingly rare, with only a few reported cases in the literature [1]. The most common isolated species is *Brevibacterium casei* [2]. There are cases of osteomyelitis, endocarditis, brain abscess, and peritonitis [3-6]. However, their involvement in CSF infections is extremely rare, and no specific guidelines exist for their management in such cases. Here, we present a case of *Brevibacterium* species in CSF, emphasizing the unique clinical features, etiology, diagnostic evaluation, and management strategies.

## Case presentation

**Figure 1:**
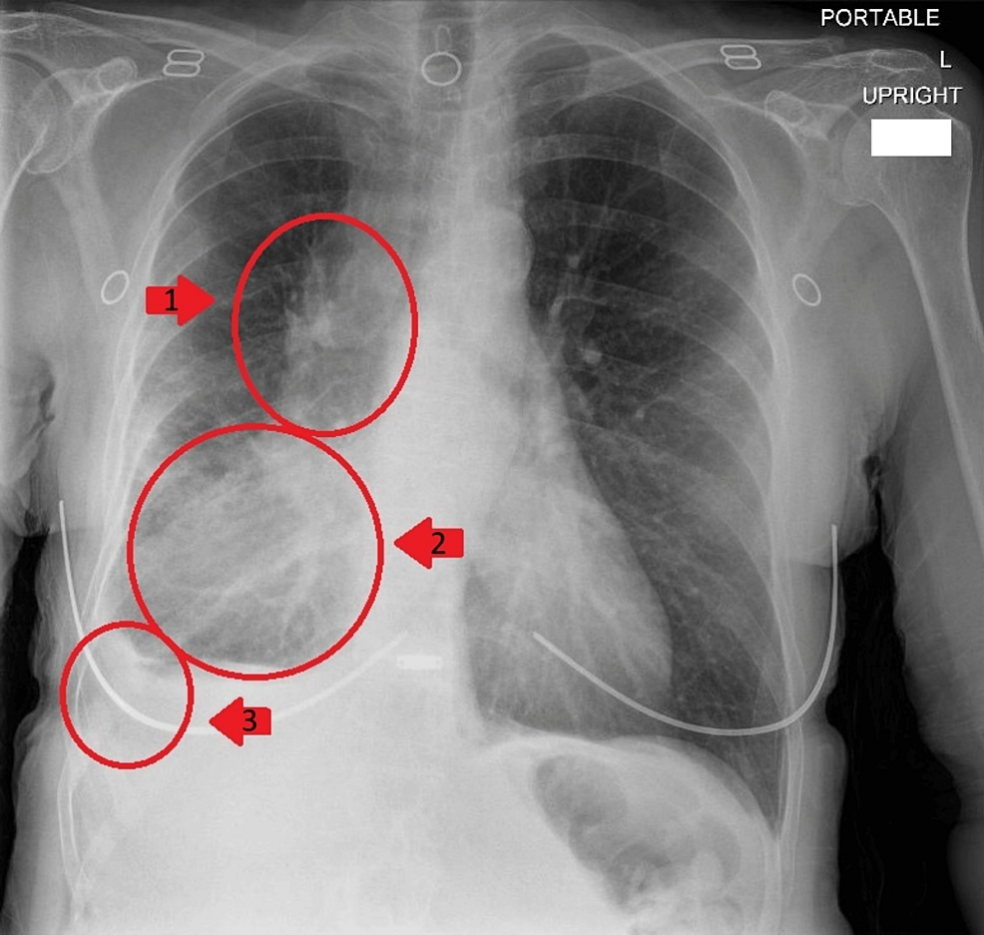
Chest X-ray revealing right middle lobe (1) and lower lobe (2) infiltrates with a small pleural effusion (3).

**Figure 2:**
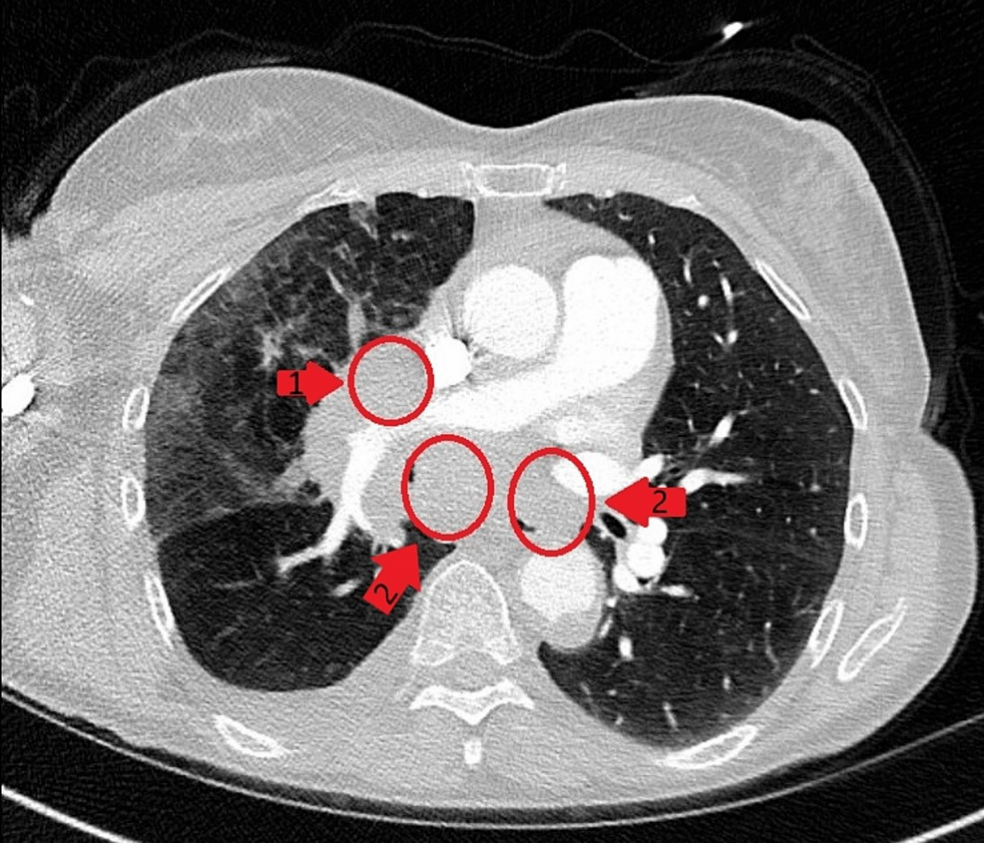
CT scan of the chest revealing right hilar adenopathy (1) and mediastinal adenopathy (2).

A 60-year-old female with a past medical history of chronic obstructive pulmonary disease, hyperlipidemia, and current methamphetamine use presented to the emergency department with worsening shortness of breath, productive cough, and brown sputum. Physical examination revealed a restless, anxious-appearing female, alert and oriented ×4, faint wheezing in all lung fields, tachycardia with regular rhythm, a left eye droop, and no neurologic deficits. Imaging studies revealed right middle and lower lobe infiltrates with a small pleural effusion, as well as mediastinal and right hilar adenopathy as seen in Figure 1 and Figure 2. Further workup led to the diagnosis of human immunodeficiency virus (HIV) infection with a low CD4 count (as seen in Table 1) and reactive serologic tests for syphilis (as seen in Table 2). A lumbar puncture demonstrated a positive venereal disease research laboratory (VDRL) in the CSF (as seen in Table 3). The patient was initiated on intravenous penicillin G for neurosyphilis treatment.

**Table 1:** Quantification of HIV-1 nucleic acid amplification test, ABS CD4 count and percentage, ABS CD3 count and percentage, and ABS CD8 count and percentage. HIV: human immunodeficiency virus; NAAT: nucleic acid amplification test; ABS: absolute

Laboratory test	Patient values	Reference range
HIV-1 Qnt NAAT	419 copies/mL	30-10,000,000 copies/mL
HIV-1 Qnt NAAT	2.62 log copies/mL	1.47-7.00 log copies/mL
% CD4	26%	25-65%
ABS CD4 T cells	56 cells/μl	396-1,309 cells/μl
% CD3	57%	57-85%
ABS CD3 T cells	123 cells/μl	723-2,271 cells/μl
% CD8	32%	20-43%
ABS CD8 T cells	71 cells/μl	224-1,014 cells/μl

**Table 2:** Syphilis Abs, IgG, IgM, and RPR titer results. Abs: antibodies; IgG: immunoglobulin G; IgM: immunoglobulin M; RPR: rapid plasma reagin

Laboratory test	Result	Reference range
Syphilis Abs, IgG/IgM	Positive, ≥1.10 units	<0.9 units
RPR	Reactive	Non-reactive
RPR titer	1:256	0-1:1000

**Table 3:** CSF studies including glucose, protein, volume, color, RBCs, WBCs, segmented neutrophils, lymphocytes, VDRL, and HIV-1 RNA QN PCR. CSF: cerebrospinal fluid; RBCs: red blood cells; WBCs: white blood cells; VDRL: venereal disease research laboratory; HIV: human immunodeficiency virus; RNA: ribosomal nucleic acid; QN: quantitative; PCR: polymerase chain reaction

CSF test	Result	Reference range
CSF glucose	82 mg/dL	50-80 mg/dL
CSF protein	23 mg/dL	15-45 mg/dL
CSF volume	10 mL	N/A
CSF color	Colorless	Colorless
CSF RBC	162 cells/UL	<1 cells/UL
CSF WBC	2 cells/UL	<5 cells/UL
CSF segmented neutrophils	19%	2%±5
CSF lymphocytes	73%	62%±34
CSF VDRL	Reactive, titer 1:4	Non-reactive
CSF HIV-1 RNA QN PCR	<20 copies/mL	30-10,000,000 copies/mL

Subsequently, a mediastinal lymph node biopsy revealed small cell carcinoma of the lung. In an unexpected finding, CSF cultures grew *Brevibacterium* species. Matrix-assisted laser desorption/ionization time-of-flight (MALDI-TOF) mass spectrometry confirmed the identification. Because meningitis was not a recognized complication of *Brevibacterium* species infection, its isolation in combination with HIV infection and malignancy prompted management with 14 days of intravenous vancomycin 1 g twice daily.

For stage 4 small cell lung carcinoma, treatment was initiated utilizing cisplatin 50 mg daily for three days, etoposide 160 mg daily for three days, and dexamethasone 10 mg daily with chemotherapy. After her first cycle of chemotherapy, subsequent assessments included repeat CD4 measurements, revealing an absolute CD4 count of 675 and a CD4% of 35%. Importantly, the patient did not have acquired immunodeficiency syndrome (AIDS).

## Discussion

The initial presence of a positive screening syphilis antibody test, along with an elevated reactive rapid plasma reagin (RPR) titer, prompted the decision to conduct a lumbar puncture to explore the possibility of neurosyphilis. The identification of *Brevibacterium* species in the CSF but not in the bloodstream prompted concerns regarding possible contamination. Nonetheless, repeated culturing of the same CSF sample and confirmation with MALDI-TOF mass spectrometry solidified the diagnosis. The patient's immunocompromised state, with concurrent HIV infection and small cell carcinoma of the lung, likely predisposed her to the isolation of *Brevibacterium* species in the CSF. 

Given the lack of standardized treatment guidelines, management decisions were made based on available evidence and expert opinion [2]. Vancomycin has been used in immunocompromised patients, particularly those with cancer and HIV, for *Brevibacterium* bacteremia [7]. Therefore, the patient received a 14-day course of intravenous vancomycin 1 g twice daily [8]. A follow-up lumbar puncture is scheduled in six months to determine the subsequent course of action in the management plan.

The presence of *Brevibacterium* species in the CSF of our patient represents a rare and unique case, given the scarcity of reported cases involving this pathogen in the central nervous system. The immunocompromised status resulting from HIV infection and stage 4 small cell carcinoma of the lung likely played a significant role in the dissemination of the pathogen to the CSF. Notably, the patient's absolute CD4 count exhibited improvement following the initial round of chemotherapy, even in the absence of antiretroviral therapy.

## Conclusions

We present an intriguing and rare case of *Brevibacterium* species isolated from the CSF of a patient newly diagnosed with HIV and stage 4 small cell lung carcinoma. The patient's compromised immune system likely made her susceptible to this unusual infection. She was treated with a 14-day course of intravenous vancomycin at 1 g twice daily. A follow-up lumbar puncture will be performed in six months to determine the next step in management. This case report aims to enrich the existing knowledge about *Brevibacterium* species in the CSF and ignite meaningful discussions among healthcare professionals regarding its management.
